# Otorhinolaryngological adverse effects of urological drugs

**DOI:** 10.1590/S1677-5538.IBJU.2021.99.06

**Published:** 2020-08-25

**Authors:** Nathalia de Paula Doyle Maia, Karen de Carvalho Lopes, Fernando Freitas Ganança

**Affiliations:** 1 Universidade Federal de São Paulo - Escola Paulista de Medicina - UNIFESP Programa de Pós-Graduação em Medicina, Otorrinolaringologia São PauloSP Brasil Programa de Pós-Graduação em Medicina, Otorrinolaringologia da Universidade Federal de São Paulo - Escola Paulista de Medicina - UNIFESP, São Paulo, SP, Brasil; 2 Universidade Federal de São Paulo - Escola Paulista de Medicina - UNIFESP Departamento de Otorrinolaringologia e Cirurgia de Cabeça e Pescoço São PauloSP Brasil Departamento de Otorrinolaringologia e Cirurgia de Cabeça e Pescoço da Universidade Federal de São Paulo - Escola Paulista de Medicina - UNIFESP, São Paulo, SP, Brasil

**Keywords:** Cholinergic Antagonists, Adrenergic alpha-1 Receptor Antagonists, Deamino Arginine Vasopressin

## Abstract

**Purpose:**

To describe the otorhinolaryngological adverse effects of the main drugs used in urological practice.

**Materials and Methods:**

A review of the scientific literature was performed using a combination of specific descriptors (side effect, adverse effect, scopolamine, sildenafil, tadalafil, vardenafil, oxybutynin, tolterodine, spironolactone, furosemide, hydrochlorothiazide, doxazosin, alfuzosin, terazosin, prazosin, tamsulosin, desmopressin) contained in publications until April 2020. Manuscripts written in English, Portuguese, and Spanish were manually selected from the title and abstract. The main drugs used in Urology were divided into five groups to describe their possible adverse effects: alpha-blockers, anticholinergics, diuretics, hormones, and phosphodiesterase inhibitors.

**Results:**

The main drugs used in Urology may cause several otorhinolaryngological adverse effects. Dizziness was most common, but dry mouth, rhinitis, nasal congestion, epistaxis, hearing loss, tinnitus, and rhinorrhea were also reported and varies among drug classes.

**Conclusions:**

Most of the drugs used in urological practice have otorhinolaryngological adverse effects. Dizziness was most common, but dry mouth, rhinitis, nasal congestion, epistaxis, hearing loss, tinnitus, and rhinorrhea were also reported. Therefore, doctors must be aware of these adverse effects to improve adherence to the treatment and to minimize damage to the health of patients.

## INTRODUCTION

The treatment of urological disorders such as urinary tract infection and erectile dysfunction has spread to several medical specialties besides Urology, including among others, general practitioners. It is important to emphasize that drugs, especially when administered orally, may have effects on other parts of the human body besides the genitourinary tract (1). In addition to reducing the adherence of patients to treatment, the adverse effects may generate serious risks to the health of patients and hinder the management of urological diseases. Researchers are always looking for new drugs with better efficacy, safety, and tolerability (2). However, doctors must know the adverse effects of drugs to contraindicate their use or at least detect them promptly aiming minimizing the damage to the patient's health. This study aims to describe the otorhinolaryngological adverse effects of the main drugs used in urological practice.

## MATERIALS AND METHODS

A review of the scientific literature was performed using a combination of specific descriptors (side effect, adverse effect, scopolamine, sildenafil, tadalafil, vardenafil, oxybutynin, tolterodine, spironolactone, furosemide, hydrochlorothiazide, doxazosin, alfuzosin, terazosin, prazosin, tamsulosin, desmopressin) contained in publications until April 2020. Manuscripts written in English, Portuguese, and Spanish were manually selected from the title and abstract. Studies that did not provide information sufficient for analysis were excluded. Additional bibliographical research was carried out to provide specific information on the drugs used in urological practice. The necessary information and data were extracted from the selected studies. The main drugs used in urology were divided into five groups: alpha-blockers, anticholinergics, diuretics, hormones, and phosphodiesterase inhibitors.

## RESULTS

### Alpha-blockers

Alpha-1 adrenergic receptor blockers (α-blockers) are widely used in the management of patients with benign prostatic hyperplasia but were originally developed for the treatment of systemic arterial hypertension (3). Examples of drugs in this group are doxazosin, terazosin, prazosin, alfuzosin, and tamsulosin. The blocking of alpha-1 adrenergic receptors in the genitourinary tract promotes relaxation of the smooth muscle of the prostate, urethra, and neck of the bladder with a consequent decrease in pressure in the prostate urethra and facilitating urinary flow (3).

Because of the distribution of alpha-1 adrenergic receptors in various organs and systems, the adverse effects that may occur during treatment with these drugs involve, but are not limited to, postural dizziness, lethargy, fatigue, fluid retention, rhinitis, sexual dysfunction, asthenia, syncope, headache, visual clouding, nasal congestion and xerostomia (3-6). Third-generation alpha-1 adrenergic blockers (alfuzosin, tamsulosin) are considered “uro-selective” and are rarely associated with adverse cardiovascular or central nervous system effects and can be safely prescribed in contrast to their predecessors (doxazosin, terazosin, prazosin) (7, 8). Dizziness and rhinitis have been described as adverse effects of tamsulosin (9).

### Anticholinergics

Scopolamine is the main non-selective antagonist drug of acetylcholine muscarinic receptors used in Urology (10, 11). In the genitourinary tract, it promotes an increase in bladder capacity, decreasing the frequency of contractions of the detrusor muscle, and delaying the initial desire to urinate (12). In otorhinolaryngology, this drug can be used transdermal in some patients to prevent and treat motion sickness (10). It is also useful in suppressing pendular nystagmus and downbeat nystagmus since the drug acts on the muscarinic receptors present in the medial vestibular nucleus, a structure responsible for maintaining the eyes in an eccentric position (13).

The main adverse effects of anticholinergics, especially scopolamine, are increased intraocular pressure, xerostomia, dizziness, visual clouding, and decreased concentration, micturition dysfunction, tachycardia, and sleepiness (14, 15). Transdermal administration of scopolamine was developed to reduce serious adverse effects and maintain the action of the drug over an extended time (approximately 72 hours), with xerostomia being the main adverse effect when administered by this route (11). After transdermal administration, adverse effects on the central nervous system (disorientation, memory disorders, dizziness, restlessness, hallucinations, confusion, and insomnia) have been reported with low frequency (16). No significant influence on the sense of smell was identified after the systemic administration of scopolamine hydrobromide (17).

Oxybutynin and tolterodine are powerful antagonists of muscle receptors (18) and have been used in the treatment of overactive bladder for over 40 years (19, 20). The adverse effects of these drugs differ little from those associated with scopolamine; xerostomia, visual clouding, constipation, erythema, fatigue, sweating, dizziness, cognitive changes, sleepiness, and urinary retention have been reported in the literature (18, 19, 21, 22). Epistaxis, characterized by the nasal-mucosa dryness as the main factor, was reported as an adverse effect of oxybutynin but was seen in one patient only (23). Facial flushing is another frequent adverse effect due to vasodilation that can play a role in triggering epistaxis (23). In comparison to oxybutynin that acts mainly on the subtypes M1 and M3 of muscle receptors and is more present in the parotid than in the bladder, tolterodine acts on subtypes M2 and M3 muscle receptors and has fewer adverse effects because it reaches the bladder more than any other areas of the body (20). The study by Paquette et al. evaluated the cognitive performance of patients who used anticholinergic drugs; dizziness was the most frequently reported adverse effect that was observed in 3% of people who used oxybutynin, 1.8% who used tolterodine, and 1.6% on placebo (24).

### Diuretics

Diuretics are widely used for the treatment of systemic arterial hypertension and edematous states. There are several classes of diuretics drugs that act through various mechanisms of action (25). There are three main classes of diuretics drugs routinely used in clinical practice that have adverse effects - loop diuretics such as furosemide, potassium-sparing diuretics such as spironolactone, and thiazide such as hydrochlorothiazide.

Furosemide is the main representative of loop diuretics (26). Common adverse effects of this class of drugs include headache, gastrointestinal disorders, hypernatremia, hypokalemia, and dehydration (27). Its otorhinolaryngological effects include hearing loss, tinnitus, and dizziness/vertigo (28). Ototoxicity is a relevant adverse effect that can be transient or permanent (29). Several investigations to elucidate the ototoxicity mechanisms of furosemide have been performed, but they are still unknown (29). Methods to avoid ototoxicity include slow and continuous infusion instead of bolus injection, use of fractionated oral doses, and even the quantification of blood levels to avoid the serum concentration of furosemide above 50µg/mL (29).

Spironolactone is a diuretic aldosterone antagonist and potassium-sparing diuretic (26). Aqueous rhinorrhea adverse effect was observed during an otorhinolaryngological physical examination that coincided with the use of spironolactone (30). The clear aspect of nasal fluid, chemo-cytological analysis, and glucose levels ruled out the differential diagnosis of cerebrospinal fluid. Moreover, rhinorrhea ceased after the suspension of the drug and recurred after repeated introduction (30). One possible explanation is that spironolactone may decrease the vascular response to norepinephrine, with the consequent nasal vasodilator effect triggering aqueous rhinorrhea (30).

Hydrochlorothiazide is the most widely used thiazide-type diuretic (31). Common adverse effects of this class of drugs include nausea, dizziness, headache, polyuria, dehydration, hyponatremia, hypokalemia, and hypomagnesemia (32).

### Hormones

Desmopressin (DDAVP) is an analog of vasopressin and is a potent agonist of the vasopressin-2 receptor (VR2) used in the management of diabetes insipidus, night enuresis, and hematological disorders (33). DDAVP increases water reabsorption by acting on the renal tubules, with the consequent increase in urinary osmolality and a decrease in urinary volume (34).

Vasopressin type 2 receptors are also found in other sites besides the kidney such as the inner ear and vascular endothelium (35). The use of desmopressin may be associated with a worsening of endolymphatic hydrops in the inner ear through the presence of VR2 in the endolymphatic sac (36-39). These receptors are also associated with increased levels of clotting factor VIII, von Willebrand factor, and tissue plasminogen activator in endothelial cells (35). A systematic review suggested the use of DDAVP in otorhinolaryngological surgical procedures as a prophylactic agent in the perioperative period and as an intervention agent in postoperative bleeding (40).

Desmopressin has few adverse effects with the most common being facial flushing. Other adverse effects include headache, decreased blood pressure, tachycardia, fatigue, and dizziness (33, 41, 42).

### Phosphodiesterase inhibitors

Initially developed for the control of systemic arterial hypertension and angina pectoris, phosphodiesterase-5 inhibitors (iPDE5) are now widely used for the management of penile erection when this action has been observed as an adverse effect of a drug (43). Sildenafil, tadalafil, and vardenafil are the main representatives of iPDE5. The most frequently reported adverse effects associated with the use of these drugs are headache, facial flushing, dyspepsia, nasal congestion, rhinitis, visual disorders, and myalgia (44). Otorhinolaryngological adverse effects associated with the use of iPDE5 include sudden hearing loss, usually unilateral, occurring in the first three days after using the drug and frequently associated with tinnitus, dizziness, or vertigo (44), epistaxis has also been observed with some patients (6).

The main otorhinolaryngological adverse effects of drugs most commonly used in urological practice are listed in Table-1.

**Table 1:** The main otorhinolaryngological adverse effects of drugs most commonly used in urological practice.

Class	Drug	Adverse effects
Alpha-blocker	Doxazosin	Rhinitis, nasal congestion, xerostomia
	Terazosin	Rhinitis, nasal congestion, xerostomia
	Prazosin	Rhinitis, nasal congestion, xerostomia
	Alfuzosin	Rhinitis, nasal congestion, xerostomia
	Tamsulosin	Dizziness, rhinitis
Anticholinergic	Scopolamine	Xerostomia, dizziness, suppression of pendular nystagmus and downbeat nystagmus
	Oxybutynin	Xerostomia, dizziness, epistaxis
	Tolterodine	Xerostomia, dizziness
Diuretic	Furosemide	Hearing loss, tinnitus, dizziness/vertigo
	Spironolactone	Rhinorrhea
	Hydrochlorothiazide	Dizziness
Hormone	Desmopressin	Endolymphatic hydrops, dizziness, procoagulant effects
Phosphodiesterase inhibitor	Sildenafil	Nasal congestion, rhinitis, sudden hearing loss, tinnitus, dizziness/vertigo, epistaxis
	Tadalafil	
	Vardenafil	

## CONCLUSIONS

Most of the drugs used in urological practice have otorhinolaryngological adverse effects. Dizziness was most common, but dry mouth, rhinitis, nasal congestion, epistaxis, hearing loss, tinnitus, and rhinorrhea were also reported. Therefore, doctors must be aware of these adverse effects and identify them early to minimize possible damage to the health of patients and poor adherence to treatments, or even contraindicate the use of some drugs.
